# An assessment of scientific and technical aspects of closed investigations of canine forensics DNA – case series from the University of California, Davis, USA

**DOI:** 10.3325/cmj.2011.52.280

**Published:** 2011-06

**Authors:** Günther Scharnhorst, Sree Kanthaswamy

**Affiliations:** 1 The Jan Bashinski DNA Laboratory, California Department of Justice, Richmond, Calif., USA; 2 Department of Environmental Toxicology, University of California – Davis, Davis, Calif., USA

## Abstract

**Aim:**

To describe and assess the scientific and technical aspects of animal forensic testing at the University of California, Davis. The findings and recommendations contained in this report are designed to assess the past, evaluate the present, and recommend reforms that will assist the animal forensic science community in providing the best possible services that comply with court standards and bear judicial scrutiny.

**Methods:**

A batch of 32 closed files of domestic dog DNA cases processed at the University of California, Davis, between August 2003 and July 2005 were reviewed in this study. The case files comprised copies of all original paperwork, copies of the cover letter or final report, laboratory notes, notes on analyses, submission forms, internal chains of custody, printed images and photocopies of evidence, as well as the administrative and technical reviews of those cases.

**Results:**

While the fundamental aspects of animal DNA testing may be reliable and acceptable, the scientific basis for forensic testing animal DNA needs to be improved substantially. In addition to a lack of standardized and validated genetic testing protocols, improvements are needed in a wide range of topics including quality assurance and quality control measures, sample handling, evidence testing, statistical analysis, and reporting.

**Conclusion:**

This review implies that although a standardized panel of short tandem repeat and mitochondrial DNA markers and publicly accessible genetic databases for canine forensic DNA analysis are already available, the persistent lack of supporting resources, including standardized quality assurance and quality control programs, still plagues the animal forensic community. This report focuses on closed cases from the period 2003-2005, but extends its scope more widely to include other animal DNA forensic testing services.

There are an estimated 72 million domestic dogs (*Canis familiaris*) in the US (1) and many dog owners share their homes with their pets. Despite the proximity of canines to humans and human activities, canine DNA evidence remains a largely untapped forensic resource even as human DNA databases are expanded (2). Aside from investigations of dog attack cases to identify the biting dog(s), canine DNA evidence can also be used in criminal investigations to demonstrate proximate associations between human suspects and human victims (3). Therefore, it is imperative to demonstrate the validity of canine DNA analysis and thus ensure the integrity of this application for the criminal justice system.

The high likelihood of finding mixtures of human and dog forensic samples in crime scenes, especially shed hairs (4), makes it extremely crucial to know the species of origin of the sample prior to assignment of samples to a particular individual by means of DNA analysis. The ability to detect (and quantify) target DNA in mixed-species samples and accurately determine the species from which the probative sample originated will help analysts minimize consumption of limited samples and efficiently optimize the genotyping tests.

Current animal forensic DNA methods and resources are not as developed as in human forensics. Of chief concern is the lack of an accredited and comprehensive Quality Assurance System (5) for animal forensic DNA testing including:

1. Quality assurance program for the systematic actions needed to demonstrate the service meets specified requirements of quality;

2. Quality control (including day-to-day operational techniques and activities to fulfill requirements for quality, a quality manual that states the policy, quality system and practices of the laboratory, and tests to measure proficiency in both technical skills and knowledge of the analysts);

3. Standard operating protocols or SOPs (for preservation and chain of custody of animal biological evidence, methods, materials, equipment and analytical procedures, and casework documentation, reporting and testimony) geared toward animal forensic laboratories.

In the US, trial court system non-human DNA evidence is not accorded the same weight as human DNA evidence and is not frequently considered as admissible. Furthermore, DNA analysis of animal evidence is as expensive and time-consuming as human DNA identification, therefore animal forensic tests are typically reserved for cases in which other forms of identification have failed or are being disputed. The technical inability to obtain meaningful information about the source of canine hair or other biological samples without resorting to specialized laboratories has also contributed to why such evidence has not being utilized to its full potential in civil and criminal investigations (6). Moreover, expertise and interest in government forensic laboratories in using animal DNA, including analysis of canine biological evidence, is still lacking.

Since 1996, DNA-based investigations involving a variety of domestic and wildlife species including cattle, horses, bears, and canines have been conducted at University of California (UC), Davis. Typical forensic cases UC Davis has been involved in can be categorized into three distinct types: 1) when the animal is the victim such as in dog abuse, theft, or killing of dogs, and dog fighting cases; 2) when the animal is a suspect, for example when a dog attacks or mauls humans or other animals; 3) when the animal is a passive witness to a crime, such as when dog hair is used to link a suspect or perpetrator to the crime scene or victim. Such cases can include arson, homicide, rape, burglary, etc (2,7,8).

The importance of forensic analysis of animal DNA at UC Davis is reflected in the range of submitting agencies/clients that include attorneys (3 cases), law enforcement or other government agencies (11 cases), private individuals (7 cases), and human medical doctors or doctors of veterinary medicine (11 cases). Cases were submitted from 15 different states in the US – Oregon (2 cases), California (7 cases), Florida (1 case), Wisconsin (3 cases), North Carolina (1 case), Kentucky (1 case), New York (4 cases), Michigan (1 case), Virginia (3 cases), Maryland (1 case), Utah (1 case), Louisiana (1 case), Alabama (2 cases), Colorado (2 cases), and Alaska (1 case). An additional case from Bermuda further demonstrates the importance of animal forensics outside of the USA. These cases have not been reported elsewhere.

To improve canine genetic testing in the US and to promote the use of canine forensic evidence in civil and criminal investigations, an affordable standardized and validated canine short tandem repeat (STR) loci reagent kit has been developed for commercialization and a publicly accessible canine STR database has been established in the US (3,6,9). To foster continued confidence among the law enforcement communities that animal forensic DNA methods are accurate, relevant, and reliable, and to further advance the quality of services provided by animal forensics laboratories, this study reviews the scientific and technical aspects of closed canine DNA cases from the UC Davis. In light of the DNA database and other published/reported findings (3,9), the review’s aim is to formulate further improvements to the animal forensic community’s ability to provide a work product and work flow that is scientifically objective and responsive to the needs of the criminal justice system.

Similar reviews of human forensic DNA analysis have led to the refinement in quality assurance/quality control (QA/QC) programs and SOPs in accordance to the FBI’s DNA Advisory Board Quality Assurance Standards (5,10,11). For example, a 2005 report by an independent investigator of the Houston Police Department’s crime laboratory observed serious problems in 43 of 135 DNA cases (32%), 4 of which resulted in death penalty sentences (12). If problems exist in human DNA laboratories, it is reasonable to expect deviations from generally accepted forensic standards in animal forensic laboratories that can undermine the reliability of animal DNA evidence. As such, analysts who use animal DNA evidence should anticipate scientifically rigorous examination of their animal forensic applications before routine admission into the trial court system and also during post-trial reviews.

## Materials and methods

A batch of 32 closed case files involving domestic dog DNA cases processed by the UC Davis Veterinary Genetics Laboratory, between August 2003 and July 2005 were reviewed in this study. The case files comprised copies of all original paperwork, copies of the cover letter or final report, laboratory notes, notes on analyses, submission forms, internal chains of custody, printed images and photocopies of evidence, and administrative and technical reviews of those cases. During the review of each case file, the QA/QC and SOP requirements for human laboratories as stipulated by the FBI’s DNA Advisory Board Quality Assurance Standards were considered (5). This review focuses primarily on aspects within the QA, QC, SOPs pertaining to sample handling, storage and integrity, casework documentation, and whether the DNA analyses, including genotyping, statistical analysis, and interpretation, are consistent with scientific conclusions in the laboratory report.

Since these cases were completed during the period between 2003 and 2005, the queries were designed to look for departures from standards in forensic DNA testing established in the 1990s and early 2000s. To perform a substantive review, the detailed checklist of queries (web extra material 1) (web extra material 1) was assembled to determine the type of evidence, the type of case (if known), who submitted the evidence, the condition of evidence seals, what testing was performed, issues involving marker selection, marker report, and allele calls vis a vis empirical support for judging the suitability of a given locus for forensic purposes, population databases used, timeframe between receipt of evidence and release of final report, etc.

## Results and Discussion

This review of 32 canine cases provided a broad perspective on the quality of animal forensic science that is seldom reported. The following is a summary of case-types:

Property damage: 2

Parentage testing: 6

Animal injury (dog or other animal)/killing or dog fighting: 7

Suspected attack by canine on another animal or person: 12

Manslaughter (dog as witness): 1

Not explicitly stated: 4

Request of submitting person/agency:

Species identification: 10

Match comparison (to evidence sample(s) or for parentage): 23 (one case requested both species identification and match comparison)

Case averages:

Evidence submitted: 3.7 samples/case

References submitted: 1.7 samples/case

Calendar days between evidence submission and release of report: 60

Testing performed:

DNA quantitation: 20

STRs used: 26

Mitochondrial DNA (mtDNA) sequencing: 10

During this review, it was observed that analysts competently produced usable genotyping results from forensic evidence, including items from difficult cases involving extractions from marginal samples such as single hairs or a swab from a dog bite mark. While the genetic testing of DNA samples would be considered sound and acceptable for basic research, there were a number of deviations from generally accepted forensic science standards for DNA analysis that could potentially undermine the reliability of the work performed. Some of the statistical analyses and reporting of results were observed not to meet certain principles for human forensics that existed at the time and posed major risks to the overall conclusions.

Before 2003, the analytical and quality control procedures employed by the reporting laboratory were not current, properly designed, and complete. SOPs for DNA analysis prior to 2003 consisted of procedures and reference materials spliced together over time without periodic reevaluation and reorganization. There were few if any technical reviews of analysts’ work, including a review of their test results, interpretation of data, and reporting prior to 2003. It is evident that some of these problems continued into the review period (2003-2005) when substantial changes to policies and practices were beginning to take shape.

From a technical standpoint, the problems detected during the review period ranged from poorly selected genetic markers and inadequate DNA databases to poor documentation and analytical and interpretive errors that could have resulted in questionable results. These weaknesses were compounded by the absence of a quality assurance program when forensic DNA work was first initiated in the mid-1990s.

Specific aspects of the DNA analysis workflow that reflected technical and/or scientific problems are described below:

1. Sample handling practices and procedures

Prior to and during the review period, tracking of submitted evidence was not done electronically. Analysts relied on a paper-based system to create the internal chain of custody. Analysts used paper and electronic forms to record evidence, sample identification information, as well as the results obtained by tests performed on each sample. The analytical files also were maintained entirely on paper and/or electronic files.

During the review period, 23 dog cases were processed for civil or criminal investigations, including parentage cases for which an estimated 98 evidence samples or items were submitted to the laboratory. Cases that were submitted without clear background information such as who collected and submitted the evidence samples, whether a file mentioned damage to property or animal attack or abuse, or whether other evidence of a crime was described in the file were not included in this number. Chains of custody for 23 of these 98 items (23.5%) indicated that evidence seals were not intact. In one case the chain of custody did not state the condition of the seal. Exactly half of these 98 evidentiary items were submitted by various law enforcement agencies, and the other half by veterinary doctors, attorneys, and private individuals. Of the 49 items submitted by law enforcement personnel, 7 (14.3% of total submitted) were received without intact seals, and the same was true for 16 (32.7%) items submitted by other individuals.

2. Evidence handling and documentation

Twenty-six of the 32 cases involved STR analysis on at least one of the samples submitted for that particular case. Unfortunately, it is impossible to provide an exact count of the number of runs in these cases because of incomplete records on many of the runs that were performed. Some marker reports were missing from the files and therefore the laboratory notes had to be cross-checked with the file contents. As a result, a conservative count based on the information in the files indicates that no fewer than 415 runs were performed in these 26 cases. A run is defined as one complete set of quality controls processed along with one or more samples. In these runs, the marker reports show that the number of failed negative PCR controls for unknown/undocumented reasons is 10 (2.4%), while 12 extraction controls failed (2.9%), and 1 positive control failed (0.2%). It is evident from the marker reports that when a control failed, samples were typically reprocessed unless adequate data were obtained from other submissions in the case. Another documentation issue involves DNA quantitation. Template DNA quantifications were performed in 20 cases, sometimes prior to STR typing, and at other times only after multiple failed attempts at STR amplification. Appropriate positive and negative controls must be run for each test or the DNA assay results may be invalid.

**Figure 1:**
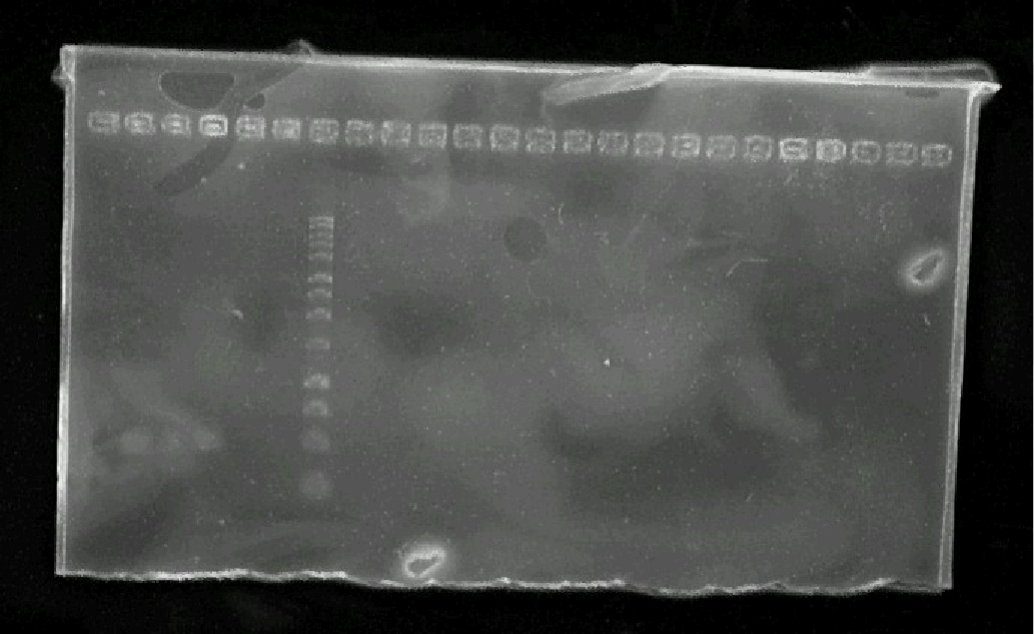
Unmodified digital image of an unlabeled yield gel from a case file.

Until March 2004, the UC Davis laboratory relied primarily on agarose yield gels to quantitate DNA and later began to use qPCR TaqMan assays to quantify template DNA in its samples. However, when yield gels were used, analysts frequently opted to provide a photograph of the gel to document the run but sometimes failed to include the quantitation estimates in the case file. Analysts also sometimes neglected to label the gel lanes with item numbers or provide a key either on the image or elsewhere in the file (Figure 1). This omission undermines the integrity of the DNA analysis. According to established standard forensic DNA procedures, the gels should be labeled to reflect the nature of the sample loaded into it, such as evidence and reference samples, sizing ladder, and negative and positive controls. Cases involving qPCR for DNA quantitation did not have this issue since the system’s software creates a results table which may be included in the case file.

The failure rates for QC were estimated to be 2.4% for negative controls, 2.9% for extraction controls, and 0.2% for positive controls. These rates appear to be unusually low, but the QA manual that was being used at the time of the testing did not specify the established interpretation thresholds (13).

3. Instrumentation and fragment sizing

During the August 2003-July 2005 timeframe, instruments used by the laboratory included the ABI Prism 377 DNA Sequencer slab gel system and the ABI 3730 Genetic Analyzer capillary system. Some of the issues involving the electrophoretic runs were specific to the slab gel electrophoresis and the single most important issue with results generated by the ABI 377 was the accuracy and precision of allele sizing due to the instrument’s gel-related problems with band resolution. Consequently, it was conventional practice to run samples in triplicate and not accept results until that level of reproducibility (consensus among all three replicates) was attained for a given locus. Unfortunately, these extra runs reduced laboratory throughput and very likely prolonged the turnaround time for these cases. The additional runs increased the cost of the analysis as well as the risk of mistakes and contamination due to processing multiple reactions. The use of slab gels also posed the risk of an increased likelihood of human errors, including pipetting errors when samples were loaded into gel lanes. Additional risks included between-lane cross-contamination and over- or under-loading.

4. Genetic markers

The laboratory used the mitochondrial Cytochrome *b* (*Cyt b*) locus for species testing based on methods described by Brodmann et al (14); for differentiating samples from closely related canines species such as domestic dogs, wolves, and coyotes, the laboratory relied on mitochondrial hypervariable 1 (HV1) sequences (15). STRs were used for the individualization of canine samples. The 29 canine-specific loci that were commonly used are provided (Table 1).

**Table 1:** The most common 29 canine short tandem repeats used by the Veterinary Genetics Laboratory in its forensics cases*

Locus	Repeat motif	Map location/linkage group	Reference(s)
AHT121	Not published	CFA 13	(16)
AHT137	Not published	CFA 11	(16)
AHTh171	Dinucleotide (GT)	CFA 6	(17)
AHTk211	Not published	N/A	N/A
AHTk253	Not published	CFA 23	(16)
C08.618	Dinucleotide (TG)	CFA 8, L 16	(18,19)
C22.279	Dinucleotide (CA)	CFA 22, L 3	(18, 20, 21)
FH2001	Tetranucleotide (GATA)	CFA 23, L 2	(16, 22, 23)
FH2054	Tetranucleotide (GATA)	CFA 12, L 6	(16, 22, 23)
FH2137	Tetranucleotide (GAAA)	CFA 3, L 3	(20, 22, 23)
FH2159	Tetranucleotide (GAAA)	CFA 24, L 7	(16, 22, 23)
FH2164	Tetranucleotide (GAAA)	CFA 6, L 14	(16, 22, 23)
FH2247	Tetranucleotide (Sequence of repeat motif not published)	N/A	(23)
FH2289	Tetranucleotide (Sequence of repeat motif not published)	CFA 27, L 26	(16, 23)
FH2305	Tetranucleotide (Sequence of repeat motif not published)	CFA 30, L 27	(16, 23)
FH2326	Tetranucleotide (Sequence of repeat motif not published)	CFA 1, L 1	(16, 23)
FH2328	Tetranucleotide (GAAA)	CFA 33	(10, 16, 23, 24)
FH2361	Tetranucleotide (GAAA)	CFA 29	(10, 16, 23)
FH2611	Tetranucleotide (GAAA)	CFA 36	(16, 25)
INRA21	Dinucleotide (TG)	CFA 21	(26)
LEI2D2	Not published	CFA 9	(16)
PEZ03	Trinucleotide (AAG)	CFA 19, L 23	(3, 18, 24)
PEZ08	Tetranucleotide (AAAT)	CFA 17, L 33	(3, 18, 24)
PEZ10	Tetranucleotide (AAAG)	CFA 14, L 25	(3, 18, 24)
PEZ11	Tetranucleotide (AAAG)	CFA 8	(3, 16)
PEZ12	Tetranucleotide (AAAG)	CFA 3, L 4	(3, 18)
PEZ18	Tetranucleotide (Sequence of repeat motif not published)	CFA 27, L 11	(16, 18)
PEZ22	Tetranucleotide (Sequence of repeat motif not published)	CFA 7, L 5	(16, 18)
RVC1	Dinucleotide (Sequence of repeat motif not published)	CFA 15	(16, 27)

*CFA – Canis familiaris autosome.

One of the most difficult aspects of developing a reliable STR multiplex panel is the selection of the proper markers. Ideal markers would produce good DNA amplifications, facilitate multiplex PCR assays, yield reliable and easy to score bands/peaks, and exhibit high heterozygosity estimates (>70%). Such highly informative markers would help minimize the total number of loci to obtain a desirable random match probability. The markers used throughout these canine cases were not originally developed for forensic purposes and their disadvantages are apparent based on the numerous occasions when evidence needed to be tested multiple times. Many of the commonly used markers often failed to amplify or were difficult to size accurately, which are factors that limited their utility for discrimination purposes. Some of the loci listed in Table 1 are dinucleotides, which would have presented issues with stutter bands and mixture analysis. This is one reason why tetranucleotide STRs are preferred in human forensic testing. Dinucleotides tend to be more polymorphic, but often exhibit significant stutter bands that make precise and accurate fragment sizing and mixture interpretation a challenge. At the other extreme, hexanucleotides tend to be easier to resolve, but these loci are less polymorphic. Many of the core structures and sequences for loci used by the laboratory have not been described sufficiently in the literature. The incorrect chromosomal map locations for loci FH2361 and FH2328 were recorded in the laboratory’s literature. However, as reported in Tom et al (9), the correct map information for these loci is presented in Table 1. It was initially hoped that it might be possible to provide empirical data on the success or failure rates for the typing of each locus in these cases, but the marker reports contained in the case files did not distinguish between whether a marker failed to amplify or was dropped from further testing for a specific case.

5. Database and profile frequencies

The true significance of a DNA match cannot be conveyed without an appropriate profile frequency and random match probability estimates to determine how rare a specific profile is in a population (4,9). The statistical meaning of a comparison between profiles developed from known reference samples and questioned samples should be based on a current and comprehensive population genetic database that represents the genetic variation of the entire population (4,9). While appropriate numbers of individual dogs per breed, from both rare and common breeds, were included in their DNA database (28), until 2003 the database did not have sufficient information on genetic variation from cross-bred (or mixed breed/outbred) dogs, which represent almost half the entire US domestic dog population (3,4,6,9).

Nonetheless, based on the casework files, it appears that it was not a standard practice to calculate profile frequency estimates during casework unless specifically requested by the case submitter. Frequency estimates were requested and provided in only 3 of the 32 cases, one of which involved both mtDNA and STR frequencies. Two of the cases either failed to produce any STR results or produced a partial profile and as such the analysts resorted to mtDNA analysis and provided the client with information on the frequency of the dog haplotypes in its canine mtDNA database. This method of mtDNA frequency estimate is referred to as the “counting method” and its statistical robustness depends entirely on the size and representativeness of the mtDNA database (29). Furthermore, because the “product rule method” is used for segregating markers and cannot be applied to estimating mtDNA haplotype frequencies (29), analysis based on mtDNA is not as discriminating as it is for STRs. In the two cases where either a full or partial STR profile was produced, frequency estimates were provided, but either clear errors were made or the methods used were not explained adequately.

One of these case files contained a table that provides the laboratory’s allele frequencies from its database of 28 American Kennel Club-recognized breeds for the AHT121 and AHT137 STR loci (the only successfully amplified loci for the questioned samples), as well as the laboratory’s calculated match probability with adjustments for genetic substructure for heterozygous loci. When the match probabilities based on these loci were re-estimated using the NRCII (29) recommendation 4.10b formula, the original estimate could only be reproduced after the θ (ie, coefficient of coancestry) value was adjusted from 0.23 (as provided in the notes) to a much more realistic 0.023. The coefficient of coancestry provides a characterization for how likely it is that a common gene was inherited due to the effects of population substructure (a concept similar to inbreeding) rather than through unrelated ancestors. Higher values indicate more substructure (29).

Case file FCD129 had different issues related to the application of statistical methods for DNA analysis. This case involved a dog suspected of attacking a horse. Blood recovered from the dog’s fur was tested for 13 equine STRs in an attempt to determine a connection between the offending dog and the horse. The apparent problem stemmed from the contradictory application of NRCII (29) recommended formulas in computing genotype frequencies for heterozygous and homozygous loci. The suspect dog was heterozygous at 9 canine loci examined and the genotype frequencies for these loci met Hardy-Weinberg equilibrium expectations (ie, using 2pq, without θ correction). The genotype frequencies for the 4 homozygous loci were calculated according to the formula, p^2^ + p(1 – p)θ, where θ = 0.02.

**Figure 2:**
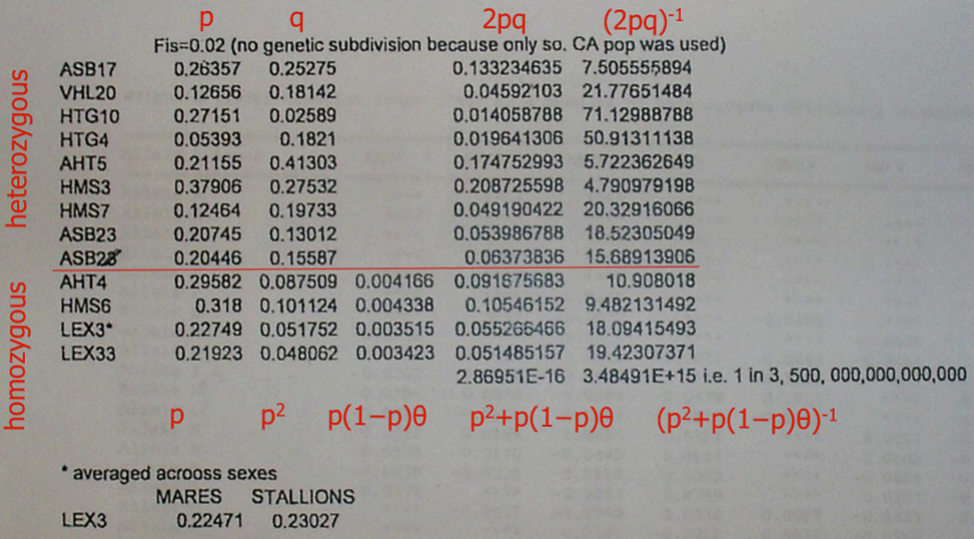
Allele frequency table from FCD129 with annotations provided by the authors.

Undoubtedly, the intention of the analysts was to provide a conservative genotype frequency in the interpretation of their DNA results. The application of a θ value to the calculation of homozygous loci frequency (ie, using the formula p^2^ + p(1 – p)θ instead of using p^2^) provides a more conservative genotype frequency estimate (ie, more common genotype). However, the use of θ to compute genotype frequencies for the heterozygous loci (ie, using 2pq(1 – θ) instead of 2pq) results in an outcome that is not conservative (ie, less common genotype). Given their commitment to being conservative in their statistical analysis, the analysts deliberately combined the relevant parts of different formulas to produce a maximally conservative estimate. However, they did not explicitly state this intention in their case folder notations. Standard methods and deviations from these standard approaches used to calculate a random match probability (RMP) should be in the SOP and do not normally need to be addressed in the report to the client. Unfortunately, the laboratory’s SOP only states, “...the report will include population genetics statistics such as allele frequencies and match probability calculations” (30) without providing specifics. As such, the results generated by this approach could have had the opposite of the intended effect by making the DNA analysis appear weak, confusing, and vulnerable to challenge. Figure 2 depicts a previously unlabeled table provided in the case file presenting the analysts’ genotype frequency estimates for this case.

6. Reporting

The laboratory’s standard terminology for its system of controls did not conform to standard reporting conventions and led to confusion during this review. The laboratory used the phrase “PCR control” instead of “amplification blank” or “PCR blank” in its marker reports. Since a control may be either positive or negative in nature this distinction is important. Positive controls were labeled as NCD900, a reference to the cell line used.

The final reporting of the cases during the review period resulted in a presentation of the forensic investigations’ results and interpretation consistent with the DNA analysis and outcomes. However, in cases involving problematic statistical analysis, the accuracy of the reports may be questionable. Also, because it is apparent that the reports were written in accordance with human forensic terminology, some of these reports appeared awkward especially when describing profile frequencies and probabilities of match comparison.

Recommendations for improving animal DNA forensic and identity testing:

1. Sample handling

An electronic case management system should be installed in the laboratory to track the status of each case submitted to the laboratory and to ensure proper handling of the samples, distribution, and statistical accounting of analytical findings. Analysts must be required to record the conditions of evidence seals when items are received. This recommendation is concordant with the International Society for Forensic Genetics requirement concerning the integrity and traceability of evidence (31).

2. Evidence testing and documentation

Unsealed samples and samples with compromised or inadequate packaging should be rejected by the laboratory and properly disposed of with supporting documentation. The submitter must be informed in written form of this disposition. Since this problem affected approximately one-fourth of all submissions, the laboratory should emphasize this to its clients before they submit the samples to ensure that the samples are properly collected and sealed (32). The best time to do this would be when potential clients first contact the laboratory to learn about the laboratory’s forensic services. An outreach initiative by the California Department of Justice’s Jan Bashinski DNA Laboratory in Richmond, CA, has resulted in a marked improvement in the quality of submissions received from law enforcement. This initiative can be emulated by the UC Davis laboratory on a smaller scale.

Analytical notes or documentation of the entire workflow during DNA analysis is necessary for supporting the scientific conclusions in the final report. Each sample should have an electronic internal chain of custody, a sample log sheet and notes made by the analysts, and digital photographs of evidentiary items and photographs of gels which should be stored electronically. Proper documentation permits effective technical and administrative reviews of the casework and allows external reviews. The documentation also facilitates the introduction of the casework into the court. Finally, it provides an audit trail that would allow assessments for continued quality of the services provided by the laboratory.

Questioned samples and reference samples should always be processed separately. Whether it is a questioned or reference sample, it is advisable that the samples with the lowest predicted DNA quantity should be tested first to prevent cross-contamination especially in smaller laboratories which lack separate areas for this purpose. In non-human DNA forensics, an analyst will examine a wide array of bio-materials that derive from various species under different environmental challenges. Therefore, these non-human forensic laboratories ought to experiment with different extraction techniques to determine the most effective techniques and optimal conditions for each situation. Techniques to improve DNA quantification should be implemented including the use of real time qPCR method (33). DNA quantity estimates and proper labeling that were frequently absent from the analysts’ notes when yield gels were used directly reflect inadequate training of the analysts. This issue may be addressed with a clearer SOP and could be alleviated by proper training documents, additional training, closer supervision, and appropriate corrective actions.

These issues also highlight deficiencies at the technical review level. Aside from performing technical reviews on all cases reported by the laboratory, it is strongly recommended that closer technical oversight and meaningful and effective technical reviews be conducted in the future to prevent the recurrence of these problems. The technical review process must ensure that appropriate analysis of the evidence was performed regarding the choice of procedure, methods and application of analytical procedures, and documentation and interpretation. The case files must be reviewed for completeness, accuracy, and details to support all conclusions. In essence, the requirement for complete and concise documentation is consistent with the requirement for maintaining compliance with “objective standards,” as embodied by the ASCLD/LAB accreditation criteria and the ISO/IEC 17025 standard, which is notable for its requirement of a documented quality management system (34).

3. Instrumentation and fragment sizing

Allelic ladders are needed for reproducibility of STR fragment sizing. Components of the allelic ladder and the sample fragments that exhibit the same sequence and length will migrate at the same rate during electrophoresis regardless of environmental changes. Species-specific internal sizing ladders need to be run during electrophoresis along with the known and evidence samples in order to permit accurate interpretation of the allelic bands or peaks. Since there are no commercially available sizing standards for animal forensic DNA testing, the laboratory needs to develop its own allelic ladders for each of the animal species it tests. Allelic ladders are pooled sets of common STR alleles present in the population of a particular species (35). These alleles can be produced using the same primers as the tested samples and therefore provide ideal sizing reference points for accurate genotyping.

4. Genetic Markers

The laboratory is encouraged to use standardized and validated markers for DNA testing. For example, multiplexed STR reagent kits for canine testing are already available commercially (3,6,9). The use of standardized panels allows comparisons of test results between laboratories assigned to the same case and facilitate review of case files by outside scientific reviewers. If a laboratory has developed markers for species and populations for which no published marker panels exist, it is recommended that the laboratory publish this information in peer-reviewed journals. Along with known and potential error rates, PCR controls and reagent recipes must be included in these publications.

5. Databases and profile frequencies

Different sample collection techniques have been used in studies involving animal samples and populations. Some of these approaches may be advantageous in studies of mutations and inheritance (36), but could be problematic for forensic investigations. In order to answer forensic questions relating to match probability estimates, for instance in canine DNA forensics, a comprehensive profile of the domestic dog’s genetic variation needs to be established and the whole dog population in the US needs to be considered. Almost half of the US domestic dog population is composed of mixed breed dogs (4). The most appropriate method to genetically characterize the US dog population is therefore by means of random sampling and the database used in probability estimates should reflect the real composition of dog breeds and populations.

An STR reference database for canine forensic testing is available at the STRBase Web site (37), which contains information from random domestic dog samples from various pure bred and mixed breed populations in the US. This sampling is also geographically representative and since it has been established that there are geographic differences in domestic dog genetic variation in the US (4), the database is highly relevant for canine cases because it largely reflects the real populations of US dogs and also the geographic stratification of the canine genetic pool. Statistical evaluations based on the frequency of the combination of genotypes over all markers in the panel (ie, the DNA profile frequency) from this database would be effective in providing statistical meaning to a DNA match between evidence and reference samples.

If the laboratory uses the above mentioned reagent kit in its canine forensic cases, and the accompanying STR database, not only will the laboratory have used a validated system of STR analysis but additionally, genetic data generated from those cases could be uploaded into the database. As the database expands in size and information, future statistical analysis for discrimination purposes will become more powerful. In species for which public databases do not exist, the laboratory is encouraged to put this information on a publicly accessible database so that the data are available to outside reviewers and for inter-laboratory validation and comparison purposes.

In human forensic DNA typing, the weight of the DNA evidence is typically reported in match probability estimates according to the FBI’s DNA Advisory Board Quality Assurance Standards requirements (5). Since there is no such requirement in animal forensic laboratories, the laboratory does not provide profile frequency estimates to its clients unless specifically requested. A match is assigned between the known and questioned samples if alleles across all loci examined are consistent. Although this approach might not seem to present a problem in animal STR typing, several factors in addition to population subdivision (as corrected for with θ) can cause non-independence among alleles and loci, including high levels of consanguineous mating, artificial selection due to breeding efforts and genetic bottlenecks, and increase the risk of assigning a match incorrectly. Therefore, the aim of canine forensic genetic testing is to allow the identification of individual dogs by matching a questioned profile with the reference profile, and eliminating individual dogs that may not have contributed to the forensic profile. The weight of the DNA match is most commonly reported in random match probability estimates; it is insufficient to only determine if one profile is consistent with another or if there is a match between the questioned and the reference profile.

The θ correction factor or coefficient of coancestry, which measures the degree of genetic subdivision (or genetic substructure) in a population, is used as a parameter in profile frequency estimates (29). This corrective factor, which is also known as Fst, allows more conservative frequencies to be calculated because it helps account for the deficiency of heterozygotes compared to what would be expected in true Hardy-Weinberg equilibrium (3). In canine cases, estimates of other fixation indices such as Fis and Fit, which measure the degree of inbreeding due to consanguineous matings and the combined effects of inbreeding and genetic subdivision, respectively, have to be accounted for as well in order to obtain an accurate reflection of the uniqueness of a canine STR profile (3). Kanthaswamy et al (3) have demonstrated that Fis was somewhat lower among breeds (0.06) than among geographical regions (0.10), while the θ estimated among the breeds was much higher (0.09) than among geographical regions (0.002). Estimates of Fit were higher among the dog breeds including mixed breed dogs (0.14) than among the four geographic regions (0.11). Similarly, Smalling et al (4) have argued that canine mtDNA HV1 single nucleotide polymorphisms contain significant regional diversity in their distributions and frequencies, demonstrating further the value of regional and mixed breed canine mtDNA databases in statistical estimations. These observations, which are in agreement with the ISFG recommendation regarding kinship factors (31), make calculating genotype frequencies correctly even more important in canines than in humans.

Finally, there should be focused training in statistics that are relevant to animal population genetics, including the estimation of allele and genotype frequencies at the breed, subspecies, and species levels, the principles of underlying random match probabilities, and the presentation of statistical data.

6. Reporting

It must be emphasized to the analysts that the average individual, including the client, trial lawyers, and members of the jury who may read a report, has little or no background in biology or genetics. Any technical terms or jargon especially those that pertain to the statistical analysis of animal DNA testing should be explained with this fact in mind. A convenient option might be for the laboratory to have a standard glossary that may be included with the report. For frequent clients such as law enforcement and attorneys, this glossary may be left out for reasons of cost-effectiveness.

Descriptions of the statistical outcomes should pertain directly to animal population genetics including individual, breed, and species testing, and not rely on the terminology developed for human-specific forensic testing. Also, coefficients for inbreeding among domestic animal breeds are much higher and have to be accounted for in estimates of match probabilities along with the coefficient of coancestry (θ) that corrects for genetic subdivision (3).

7. Methodology validation

The canine cases give a glimpse of the different scientific and technical applications of species-specific genetic markers that are still suboptimal relative to existing generally accepted forensic principles. This necessitates well-organized and well-documented validation steps for the array of forensic DNA services this laboratory provides. Given the ultimate implications of scientific evidence that are presented in a court of law, it is highly recommended that techniques used to produce or analyze the evidence be shown as scientifically valid regardless of the admissibility standard employed by the court. This means that techniques like the amplification and analysis of animal STRs must undergo validation studies. Validation has three purposes: 1) assess the ability of defined procedures to reliably obtain the expected result, 2) to determine the limitations of the analytical procedure, and 3) to identify aspects that must be monitored and controlled (5,11).

This review was based on casework from 2003-2005 and only reflects the status of animal forensic testing during that narrow timeframe. Therefore, the review does not cover changes in policies and practices related to the current management and administration of the UC Davis laboratory’s forensic operations after that period. Since then, the laboratory has undergone advances and upgrades in its laboratory techniques. For example, as early as mid-2003, an ABI 3730 Genetic Analyzer was purchased and new training programs were initiated by late 2003. Furthermore, since 2005, the laboratory has revised its SOPs and implemented a new QA/QC program for its forensic work and in 2010 it became an American Society of Crime Laboratory Directors/Laboratory Accreditation Board accredited laboratory.

Nonetheless, in concluding this review several observations need to be made regarding animal forensic testing as many of the issues highlighted in this report remain unchanged. For instance, forensic approaches of the different applications of species-specific genetic markers are still insufficiently established for the suite of animal species being tested in the laboratory. Since the first successful use of feline STRs in genetic individualization of domestic cat hairs for a murder investigation (38), the published literature has grown dramatically on the topic of animal forensic DNA testing and its use in civil and criminal investigations. While the foundational aspects of animal DNA testing for basic research may be reliable and acceptable, the scientific basis for forensic testing animal DNA needs to be improved significantly. Apart from a lack of standardized and validated genetic testing protocols, areas needing improvement span a wide range of topics including QA and QC measures, sample handling, evidence testing, statistical analysis, and reporting (31,39).

The issues discussed are probably not specific to UC Davis, and can provide lessons for other laboratories conducting animal forensic work. An ISFG study involving 21 Spanish and Portuguese forensic laboratories showed that up to one third of these laboratories failed to obtain any mtDNA results from canine hair revealing a lack of training and several technical shortcomings (40). Animal forensic analysts should employ methodologies that follow principles of scientific methods complete with empirical testing using appropriate standards and controls and known or potential error rates. These techniques should be subjected to independent verification and peer review and publication. Because animal DNA evidence can be presented in a court of law, it should be legally defensible. It is strongly recommended that this evidence should meet the Daubert threshold of admissibility standards for reliability, robustness, and reproducibility (29).

The primary limitation of this study is its narrow time frame and timeliness. However, the review could only be based on case files that were made available to the authors and also at the time of the review these cases needed to be either settled or resolved. Objective reviews on actual casework such as this are rare and usually conducted after a court order or for a trial. Besides directly examining and assessing the scientific and technical aspects of animal forensic DNA analysis and demonstrating that the fundamental aspects of this testing are reliable/acceptable, the review also revealed several significant lapses including the lack of standardization, validated protocols, and QA/QC measures (for example, evidence contained in unsealed bags, improper controls, lack of appropriate quantification methods, problems with marker selection and allele assignment, and lack of interpretation guidelines and representative databases). Most if not all of these issues still occur in non-human forensic laboratories as described recently in van Asch et al (40) and Linacre et al (31), despite being first broached in Budowle et al (39). Therefore, despite its narrow and “dated” time frame, this unbiased, hands-on review of actual workflows and closed case files in a prominent laboratory is pertinent for improving the quality of non-human forensic DNA analysis.
