# Supplementary material for: An assessment of scientific and technical aspects of closed investigations of canine forensics DNA – case series from the University of California, Davis, USA
Source: Croat Med J. 2011 Jun;52(3):280–92. doi: 10.3325/cmj.2011.52.280 (PMC3118728; doi:10.3325/cmj.2011.52.280)
Supplement: Supplementary Table 1 [file CroatMedJ_52_s002.pdf]

Appendix 1

Checklist* of queries used to facilitate this review:

Offense or complaint:

What was requested by submitting agency?

Nature of Evidence:

How many evidence samples submitted?

How many reference samples submitted?

Date evidence submitted:

Date report released:

Elapsed time:

> Did analyst adequately describe evidence item(s)?
>
> Species testing performed?
>
> Indicate test used:
>
> CyB
>
> Immunological tests
>
> Result of species test
>
> Appropriate control samples run?
>
> Controls OK?
>
> DNA quantitated?
>
> Method used:
>
> Quant Result
>
> Appropriate control samples run?
>
> Controls OK?

STRs markers used?

STR results obtained?

Nature of STR results: Full profile, partial profile, No results

Quality of STR results:

Controls used?

List controls:

Controls OK?

Replicates run?

Replicate results match?

Loci used

mtDNA used?

MtDNA results obtained?

Forward & reverse sequencing performed?

Forward & reverse sequencing matches

Quality of sequence obtained

Reference sequence used

Mt DNA results: (e.g. "matched known reference samples")

Substrate samples evaluated

See above

If both STR loci & mtDNA typing methods used, were results concordant with one another?

See above

If more than one evidence sample was typed, were the typing results concordant?

If the typing results were not concordant, can this be explained?

Which population database(s) used?

Which database

Report: Date ____ Owner ____ Address: ____ Case # ____

Submitter's Case # ____ # samples:________ Type of match/parentage:______ # and type DNA markers:_____ DNA profile on marker report = excel spreadsheet:_____

Marker report consistent with final case report:____ Sufficient replicates run on each sample and locus:____ All replicates match:____

Date Rec'd ____ Re line _____ Spell ____

Allele Freq. ____ MP Θ _____ MP Calc. ____

Header ____

Did data in notes support conclusion(s) in report?

Were notes legible & sufficiently detailed?

Were conclusion(s) appropriately qualified?

Were frequency estimates checked & accurate?

Was report understandable to lay person?

Did the laboratory report answer the question of the submitting agency?

*This checklist was adapted from the laboratory's technical and administrative review forms.
